# Comparison of Sympathetic Skin Response (SSR) between Electrical and Acoustic Stimuli in a Healthy Pediatric Population

**DOI:** 10.3390/pediatric13030060

**Published:** 2021-09-01

**Authors:** Sara Cavaliere, Giovanna Bertini, Cesarina Cossu, Maria Bastianelli, Simonetta Gabbanini, Cristina Mei, Silvia Lori

**Affiliations:** 1 Division of Neonatology, Careggi University Hospital, 50134 Florence, Italy; saracavaliere@outlook.com; 2 Neurophysiology Unit, Neuro-Musculo-Skeletal Department, Careggi University Hospital, 50134 Florence, Italy; cossuc@aou-careggi.toscana.it (C.C.); bastianelli_me@hotmail.com (M.B.); simonettagabbanini@virgilio.it (S.G.); cristina.mei@unifi.it (C.M.)

**Keywords:** skin impedance, skin impedance potential, children, pediatric population, SSR, galvanic response

## Abstract

Data in the literature report that latency and morphology in the cutaneous sympathetic skin response (SSR) do not change according to the type of stimulus delivered, unlike the amplitude which shows greater values in relation to the intensity of the physical impact caused in patient. Since the acoustic stimulus represents a method better tolerated by the pediatric patient, the aim of this study is to evaluate the presence or absence of significant differences in SSR between electrical and acoustic stimuli. The SSR was performed for each child of 18 recruited in this study, deriving from the palm of the hand and the sole of the foot and initially delivering an electrical stimulus at the level of the median nerve at the wrist. Two acoustic stimuli were subsequently delivered with the aid of audiometric headphones. Our results show no significant differences for the amplitude values obtained (*p* values > 0.05). For the latency there was a statistically significant difference (*p*-value = 0.001) for the left hand, subsequently not confirmed by the comparison performed between the two sides (*p*-values = 0.28 and 0.56). If these preliminary data are confirmed by a larger sample, the acoustic stimulus could be introduced in a standardized protocol for performing SSR in pediatric patients.

## 1. Introduction

The sympathetic skin response (SSR) represents a neurophysiological investigation used in the functional evaluation of the sudomotor activity related to the sympathetic branch of the autonomic nervous system. This response is characterized by a potential that reflects the change in skin impedance due to the sympathetic response to a stimulus. 

For the first time, this phenomenon was described in 1890 by Tarchanoff, who associated the change in potential with a modification in the secretory activity of the sweat glands, regardless of the vascular reaction [1].

The SSR recording method was introduced into clinical practice in neurophysiology laboratories by Shahani in 1984 [2] and subsequently by Knezevic and Bajada in 1985 [3].

The method involves the use of surface pregellated electrodes placed at the palmar and plantar level, that are the registration site par excellence, since they are the sites of greatest density of sweat glands. 

Nevertheless, SSR can be recorded from any skin area, as described in 2017 by Bianchi et al., on the pelvic floor study: In this case the response is derived at the perineal level [4].

This response is produced by the synchronous activation of the eccrine sweat glands by sympathetic efferent impulses and is evoked by exteroceptive and enteroceptive stimuli.

The “emotional” sweating, which is the focus of this response’s evocation system, especially evident at the palmar and plantar level, is in part functionally independent of the thermoregulatory sweating. Its control is integrated with emotional, cognitive and neuroendocrine functioning and carried out at multiple levels within the central nervous system.

It is a polysynaptic reflex, the afferent branch is variable and is related to the type of stimulus delivered. Myelinated sensitive fibers Aδ and B and small caliber myelinated fibers are activated in a nonspecific way through somatoesthetic stimuli. These sensory afferents then reach the hypothalamus where the central processing of the stimulus takes place, by circuits mainly represented by brain structures under the influence of cognitive and emotional activity [5,6,7]. 

The efferent component of the reflex consists of sympathetic-cholinergic fibers originating from the paravertebral sympathetic chain, up to the sweat glands. With somatoesthetic stimuli, the efferent component activated is represented by the unmyelinated type C fibers, which carry the response slowly. That fibers originate from the sympathetic ganglia and then join with the peripheral nerves.

On the other hand, the acoustic stimulus activates, as the afferent branch, directly the eighth cranial nerve, the acoustic nerve, and specifically the nerve cells present in the spiral ganglion. The acoustic stimulus processing runs through the numerous projections of the geniculate nucleus towards subcortical areas, such as the central and lateral amygdala, the ventromedial hypothalamus, and the putamen [8].

At the cortical level, the anterior cingulate cortex (ACC) is involved in the control of emotional sweating and is also responsible for the habituation phenomenon, or the inhibition of the attention of redundant (reverberant) stimuli [9]. The nature of this phenomenon is still not completely clear, but the literature accepts the hypothesis that it is a cognitive adaptation to the stimulus for the reduction of attention levels [10].

The delivery of surprise stimuli, which; therefore, remedy the habituation problem, induces in the patient a change in skin impedance, which is recorded by the surface electrodes as a potential difference. This potential results as a wave of which parameters being evaluated are presence/absence, latency, and amplitude.

The unmyelinated fibers of the efferent component are those that make a greater contribution to the latency value, but even a slow conduction in the afferent branch of the reflex arc, or a central delay in the activation of sympathetic neurons, can cause relevant changes. The amplitude value is instead determined by the amount of fibers that participate in the response and the intensity of the physical impact of the stimulus that originates it. However, the presence or absence of the response is decisive.

In adults, there are numerous applications of this investigation in evaluating autonomic functionality in the context of central and peripheral pathologies.

The literature reports the use of SSR in the evaluation of autonomic deficit in 66–83% of diabetic patients presenting with peripheral neuropathies [2,10,11,12].

As regards pathologies involving the central nervous system, the literature studies on Parkinson’s disease have shown both qualitative and quantitative anomalies in the SSR response, including total loss of response [13,14,15,16,17].

Clinical studies show abnormalities in SSR in more than 50% of patients with multiple sclerosis [18,19]. SSR is also absent in 40% of 25 patients with amyotrophic lateral sclerosis (ALS) [20], while it is pathological in cases of cervical myelopathy [21] and syringomyelia [22].

Alterations of the autonomic nervous system can be also found in pediatric age in relation to central and peripheral pathologies, as evidenced by some studies.

As in adults, peripheral neuropathies can also affect pediatric age, often due to genetic mutations or inherited forms, such as in hereditary sensory and motor neuropathy type I (HMNS I) and in hereditary sensory autonomic neuropathy type IV (HSNA IV) [23,24,25].

A further alteration in the cutaneous sympathetic response in pediatric patients is observable in cases of neuromuscular diseases such as spinal muscular atrophy, as emerged from the study conducted by Hidee Arai et al. in 2005, through the evaluation of cold-induced vasodilation in the fingers and the contextual SSR with electrical and acoustic stimuli [26].

Considering that electrical stimulation represents a troublesome method for the patient, especially in the pediatric setting, if the SSR response from acoustic stimulation does not show significant differences, it could be introduced into clinical practice. The literature provides an already evaluated comparison of these two types of stimulus in a healthy young population, which shows their compatibility [27]. The aim of the present study is; therefore, to compare the SSR response obtained through the delivery of electrical and acoustic stimuli in a healthy pediatric population.

## 2. Materials and Methods

This study enrolled 18 healthy pediatric patients: Fourteen children (6 males and 8 females, mean age 10 years) and 4 infants (mean age 4 months).

Newborns were examined come from the nursery of the neonatal ward of the Careggi University Hospital.

The pediatric subjects were voluntarily recruited at the Neurophysiopathology laboratory of the Careggi University Hospital.

The materials used for registration of the SSR exam are:

- Disposable pregellated electrodes
- Electroconducting paste
- Abrasive paste
- Pediatric fork stimulator
- Audiometric headphones

The electromedical device used for recording is the Medelec Synergy EMG (ViasysTM Healthcare, Surrey, UK), with the following recording parameters: Sensitivity of 500–200 µV, bandwidth of 0.1 Hz–2 KHz, time sweep of 10 s.

### 2.1. Procedures

To perform the electrophysiological examination, children’s skin was carefully cleaned and degreased to facilitate contact with the recording electrodes, in order to reduce the ion or other impedance, which is a possible source of artifacts.

The SSR recording was performed using disposable pregellated surface electrodes. The active ones were positioned on the palm of the hand and on the sole of the foot, while the reference ones on the corresponding back side. A ground electrode was placed between the registering electrodes and the stimulation site.

A fork stimulator with reduced interelectrode distance according to the small size of the pediatric patient was used for the delivery of the electrical stimulus. In order to obtain a better propagation of the stimulus, a minimum quantity of electroconductive paste was placed on the stimulator. The stimulation site is located on the wrist at the median nerve.

The intensity of the stimulus varies from 50 to 100 mA, to ensure the effective evocation of the response, while the duration of the stimulus was 0.5 millisec. The stimulus was delivered without the patient being warned to avoid the phenomenon of habituation.

After response recording with electrical stimulus, SSR was performed through two acoustic stimuli (“tonal click”) with an intensity of 110 dB and a duration of 0.1 millisec, with the aid of headphones. The acoustic stimulus was given a few minutes later the electrical one in order to reestablish the initial autonomic equilibrium conditions.

For greater patient compliance and therefore better recording quality, the examination was explained to the child in a clear and reassuring way, especially the importance of remaining still following the stimulus so as not to affect the response latency. For the newborn, the examination procedure was explained to the parents, who were provided with a written information in which all the specifics of the examination were explained.

### 2.2. Statistical Analysis

The clinical characteristics of this study population are reported as the mean, median, standard deviation, upper and lower confidence values, and maximum and minimum values.

Since the data collected for both stimulations are related to the same group of patients, they were considered paired data.

For the study of the normality data distribution, the values of kurtosis and skewness (or asymmetry) were calculated, subsequently confirmed by the statistical Shapiro–Wilk test [28]. Data showing a Shapiro *p*-value > 0.05 were found to have a statistically normal distribution.

For data with non-normal distribution, the Wilcoxon statistical test [29], a non-parametric test for dependent data, was used. For comparisons showing a *p*-value < 0.05 (a_adjusted_ = 0.005 for multiple comparisons obtained with Bonferroni method), statistically significant differences were considered.

For data with normal distribution, the parametric *t*-test paired test was used [30].

Again, *p*-values < 0.05 (a_adjusted_ = 0.005) were considered statistically significant.

For data showing a statistically significant difference, a second comparison was performed in order to highlight possible interleaved differences in the two types of stimulus. Again, *t*-tests were used for the sample with normal distribution and Wilcoxon for the sample with non-normal distribution.

Statistical box plot and linear graphs were created to highlight the presence or absence of graphically verifiable differences.

## 3. Results

The overall 200 data obtained from the 18 pediatric patients recruited were analyzed (Table 1 and Table 2).

The sample relating to the amplitude of the right hand for electrical stimulus, (*p*-value = 0.03), of right foot for electrical stimulus (*p*-value = 0.004), of right and left foot by acoustic stimulus (*p*-value of 0.03 and 0.02 respectively) show non normal distribution. The sample with non-normal distribution for latency resulted only for the right hand for acoustic stimulus (*p*-value = 0.003). All other samples showed normal distribution with *p*-value > 0.05.

**Figure 1:**
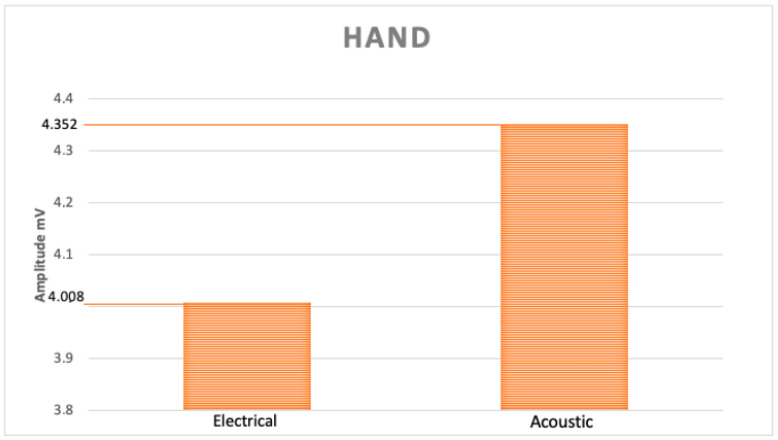
Graphic illustration of medium amplitude values of both stimuli.

**Figure 2:**
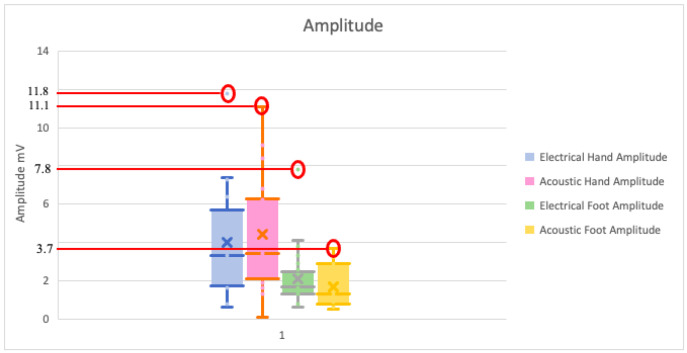
Box plot of amplitude values. Maximum values are circled in red.

Regarding the amplitude values, descriptive statistical analysis highlights that the average value obtained with electrical stimulus in the hand is lower than that obtained with acoustic stimulus (Figure 1); however, the maximum value is recorded with the electrical one (Figure 2).

For the comparison of the right hand electrical vs. acoustic stimulus, right foot and left foot, the Wilcoxon statistical test did not show significant differences, with *p*-values of 0.46, 0.84, and 0.80, respectively. For the comparison of left hand electrical vs. acoustic stimulus, having normal distribution, the *t*-test for paired data showed no statistically significant differences, with *p*-value 0.35. 

**Figure 3:**
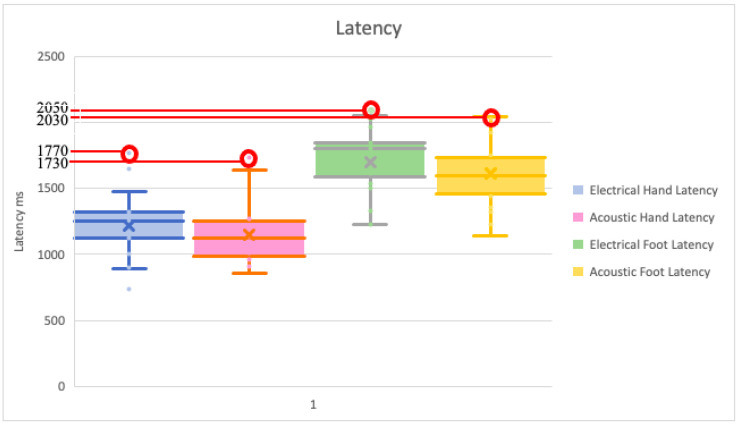
Box plot of latency values with both type of stimulus, in hand and foot. Maximum values are circled in red.

Regarding the latency values, the maximum values result for electrical stimulus (Figure 3). For the comparison of the right hand electric stimulus vs. acoustic stimulus, the Wilcoxon test did not show any differences, with a *p*-value equal to 0.66. The *t*-test for paired data for right and left foot for electrical vs. acoustic stimulus showed no differences with *p*-values of 0.25 and 0.07, respectively. Instead, for the comparison of the left hand for electrical vs. acoustic stimulus, which shows a *p*-value of 0.001, we found to be statistically significant difference.

In order to confirm or exclude differences between two sides that could justify this statistical difference, a further comparison was made for both the electrical stimulus and the acoustic stimulus, in parallel.

**Figure 4:**
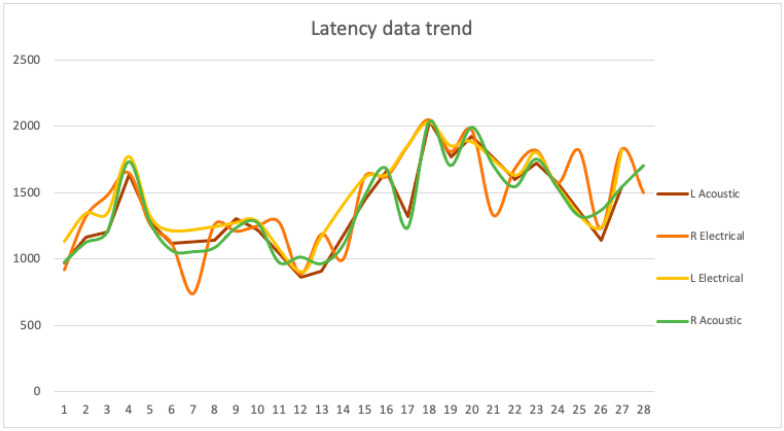
Latency values trend of both sides.

In the comparison between the two sides, no significant differences emerged, with *p*-values equal to 0.28 and 0.56 (Figure 4).

## 4. Discussion

The literature has numerous data in which the possible differences that may arise in the response from the delivery of two different types of stimuli have already been evaluated. In fact, the acoustic stimulus differs from the electrical one since it is a central stimulus, more specifically, supraspinal, while the second is characterized as a peripheral non-specific stimulus, in our case at the level of the median nerve. Reitz et al., in 2002, had already noted the similarity in the morphology and latencies of the two responses, a finding that suggested the presence of a single descending pathway in the sudomotor response, starting from the centers located at higher levels up to the spinal level and limbs [31].

This data confirmed the previous studies conducted by Akyuz et al. in 1999, Cariga et al. in 2001, Elie and Guiheneuc in 1990, Opsomer et al. in 1996, and Uncini et al. in 1988 [32,33,34,35,36], who already argued that the elicitation of the SSR response in adults was independent of the type and location of stimulation.

In this study, the maximum latency value results for electrical stimulus, albeit with a negligible difference from the maximum value obtained for acoustic stimulus (1770 vs. 1730 ms in hand, 2050 vs. 2030 ms in foot) (Figure 3). 

Statistical analysis subsequently shows a significant difference for the left hand between electrical stimulus and acoustic stimulus with a *p*-value of 0.001.

To confirm or exclude a difference in the response due to laterality, we performed two statistical tests that compared the two sides for both electrical and acoustic stimuli. There are no significant differences, confirming what the literature reports to date (Figure 2).

Otherwise, with regard to amplitude, the data in the literature suggest that there are differences in the maximum amplitude values between acoustic stimulus, electrical stimulus, and magnetic stimulus.

In fact, in the 2012 study conducted by Toyokura et al., it emerges that, while delivering an acoustic stimulus at a high intensity, to guarantee the stimulation of as many fibers as possible and; therefore, a physical impact of the stimulus that could equal the electrical one [34,37,38], the maximum amplitude value was; however, lower than the values obtained with an electrical stimulus [39]. This data; therefore, confirm that the different perception and physical impact of an acoustic stimulus arouses from that of electrical stimulus, and consequently a lower elicitation of the autonomic reflex is generated. The amplitude, in fact, corresponds to the amount of nerve fibers that participate in the response. A further confirmation of this point is represented by Hoeldtke et al. in 1992, who evaluated that, only in the presence of stimuli with a greater impact, it was also possible to minimize the inhibitory effect of habituation [40].

With our data, although the descriptive statistics show that the average value of the amplitudes obtained by electric stimulus in the hands is lower than that obtained by acoustic stimulus (4.008 vs. 4.352 mV) (Figure 1), the highest maximum amplitude value is recorded by electrical stimulus (11.8 mV in the hands, 7.8 in the feet), both for the hands and for the feet (Figure 2). Furthermore, in support of this finding, the analysis performed with statistical tests does not show any significant differences.

As a possible explanation of the average value for acoustic stimulus greater than that obtained for electrical stimulus, it is important to note that the stimulus intensity delivered in this study is 5 dB (110 dB) higher than that used by Toyokura in his study. It is also necessary to consider that the pediatric patient, especially the neonatal patient, for greater compliance, was placed in conditions of acoustic isolation, to prevent the acoustic pollution present in the ward from affecting the response. This could also have favored an initial fall asleep and, therefore, it would explain how the physical impact of the acoustic stimulus has achieved greater effectiveness.

As regards the morphology of the responses, we can consider as a starting point the classification of the responses described by Tokoyura in 1998 [9], which shows the distinction in the P-type wave, with a prevalence of the positive component, and in the N-type wave, with a prevalence of the negative component.

As mentioned before, Reitz also argued for similarity in morphology, as well as in latency, by examining the quantity and number of phases present in the response. This is because, for the author of the study, the number of phases is more relevant than the size of each component [31]. Other authors deny this finding, arguing that the appearance of a P-type morphology (i.e., with a prevalence of the positive component) is less frequent due to acoustic stimuli, since that morphology is more associated with stimuli that evoke greater impact [37,38]. In addition, the author highlights how a P-type morphology is larger than an N-type morphology, that is, with a predominantly negative component.

Our data regarding the morphology did not allow us to establish an effective correlation.

In conclusion, it is certainly of considerable importance to ensure that, from the peripheral level, there are no pathologies that could interfere with the conduction of the acoustic stimulus and that could; therefore, result in an absent cutaneous sympathetic response. Not due to the absence of the response itself, but to a peripheral inefficiency of the stimulus, which would not be able to reach the processing centers.

Since it is a method that involves the use of an acoustic stimulus, the idea of involving an otolaryngologist could be considered. Nevertheless, given that this method is often present in clinical practice, it would become too complex to involve more medical figures from a purely organizational point of view. Furthermore, the potential obtained is of neurophysiological relevance.

Nonetheless, aware of the fact that our patients recruited in the study were healthy and, therefore, did not present any type of hearing disorder, it would be useful and recommended to perform an acoustic threshold check before the method object of this study, to exclude the presence of peripheral type impediments.

To control the acoustic threshold, the same audiometric headphones would be used with the same stimulus, starting from a higher intensity and going down in intensity until the child reports that he no longer feels the stimulus (usually <35 db).

For infants, on the other hand, the method to be used would be a second one, for which, with the same audiometric headphones, stimuli are delivered as if a BAEP (brainstem auditory evoked potential) were being performed: When the V component disappears (the first to appear with the increase of the stimulus and the last one to disappear as the stimulus decreases), it means that the newborn is unable to feel the stimulus anymore.

If there was a suspicion between peripheral or central damage, the question could be investigated by performing a BAEP and be sure to exclude any impediment.

If the functionality of the ear is altered, obviously this method would be considered unsuitable.

### Limitations

The small sample size in the present study could be a limiting factor. In relation to this factor, the impossible distinction between the neonatal and pediatric population meant that normative data classified by age was not able to be provided.

## 5. Conclusions

Our results do not show statistically significant differences between electrical stimulus and acoustic stimulus in a healthy pediatric population, and represent preliminary data which, if confirmed, could allow the introduction of the acoustic stimulus in a standardized protocol for the SSR execution.

In this way, especially in the pediatric field, it would be possible to avoid the use of the electrical stimulus and thus introduce the acoustic stimulus, which is certainly better tolerated by pediatric patients.

## Figures and Tables

**Table 1:** Statistical descriptive analysis of amplitude values.

Amplitude (mV)	Hand				Foot			
	Electrical Stimulus		Acoustical Stimulus		Electrical Stimulus		Acoustical Stimulus	
	Left	Right	Left	Right	Left	Right	Left	Right
Maximum	7.4	11.8	11.1	9.1	4.10	7.8	3.7	3.4
Minimum	1.6	0.6	1.3	0.1	0.8	0.6	0.6	0.5
Mean	4.25	3.82	5.0	3.8	1.93	2.26	1.75	1.66
Median	4.50	3.00	4.90	2.90	1.70	1.70	1.30	1.25
Standard Deviation	2.21	2.98	3.33	2.59	1.00	1.81	1.16	1.03
Lower confidence value	1.6	0.6	1.3	0.1	0.8	0.6	0.6	0.5
Upper confidence value	7.4	11.8	11.1	9.1	4.10	7.8	3.7	3.4

**Table 2:** Statistical descriptive analysis of latency values.

Latency (ms)	Hand				Foot			
	Electrical Stimulus		Acoustical Stimulus		Electrical Stimulus		Acoustical Stimulus	
	Left	Right	Left	Right	Left	Right	Left	Right
Maximum	1770	1650	1640	1730	2030	2050	2040	2030
Minimum	1130	740	970	970	1540	1330	1320	1230
Mean	1322.22	1226.36	1224.44	1177.27	1757.78	1740	1671.11	1630.91
Median	1280	1260	1200	1120	1800	1810	1660	1680
Standard Deviation	180.95	245	183.04	213.22	161.92	202.04	224.25	248.21
Lower confidence value	1130	740	970	970	1540	1330	1320	1230
Upper confidence value	1770	1650	1640	1730	2030	2050	2040	2030

## Data Availability

All data are available from the corresponding author upon reasonable requests.
